# Effects of Sleep and Age on Prospective Memory Consolidation: A Walk in a Virtual Museum

**DOI:** 10.3390/clockssleep1030028

**Published:** 2019-07-17

**Authors:** Stéphane Rehel, Nicolas Legrand, Grégory Lecouvey, Alice Laniepce, Françoise Bertran, Philippe Fleury, Sophie Madeleine, Francis Eustache, Béatrice Desgranges, Géraldine Rauchs

**Affiliations:** 1 Normandie Univ, UNICAEN, PSL Université Paris, EPHE, INSERM, U1077, CHU de Caen, Neuropsychologie et Imagerie de la Mémoire Humaine, GIP Cyceron, 14000 Caen, France; 2 Unité d’exploration et de traitement des troubles du sommeil, CHU, 14000 Caen, France; 3 Centre Interdisciplinaire de Réalité Virtuelle, UNICAEN, 14000 Caen, France

**Keywords:** sleep, prospective memory, ageing, virtual reality, memory schemas, cognitive reserve

## Abstract

Prospective memory (PM) refers to our ability to perform actions at the appropriate moment, either when a predetermined event occurs (event-based, EB) or after a predetermined amount of time (time-based, TB). Sleep favors the consolidation of both EB and TB intentions, but whether this benefit is preserved during ageing is still subject to debate. PM was assessed in 28 young and 27 older healthy volunteers using a virtual environment. Participants had to learn and execute intentions after intervals filled with either daytime wakefulness or nighttime sleep. Intentions consisted of four TB, four EB with a strong link between the cue triggering retrieval and the action to be performed (EB-link) and four with no link (EB-nolink). PM was not affected by age, whatever the type of intention and the nature of the retention interval. While sleep reinforced all types of intentions in young participants, this benefit was only observed for TB and EB-link intentions in older adults. Sleep also reinforced the intrinsic PM components in both groups. Thus, when assessed using complex realistic situations, PM is not impaired in ageing. Results are discussed in the light of memory schema theory and the possible impact of cognitive reserve on sleep and memory.

## 1. Introduction

Sleep favors the consolidation of recently acquired information in long-term memory stores [1,2]. This effect has been widely reported for memories of past events, but other studies suggest that sleep may preferentially strengthen memories that are relevant for future behaviors, if they are either required for a later memory test [3,4,5] or associated with an anticipated reward [6]. Prospective memory (PM), which refers to the ability to remember to perform intended actions in the future [7], is a typical future-oriented memory process. In the past few years, studies have shown that sleep benefits PM performance [8,9,10,11,12,13,14,15], an effect mainly attributable to slow-wave sleep (SWS) [9] and mediated by spontaneous associative retrieval processes during PM recall [10].

Two essential components must be remembered in order to correctly execute delayed intentions [16]. The *prospective* component involves remembering that something has to be done at the appropriate moment, while the *retrospective* component refers to the content of the intention (i.e., remembering what has to be done). The retrieval of an intention can be triggered either by the occurrence of an external event, namely the prospective cue (e.g., “take the cake out of the oven when the timer rings”) or after a defined amount of time (e.g., “take the cake out of the oven after 30 minutes”). These two forms of PM refer respectively to event-based (EB) and time-based (TB) PM [16]. While these intentions are held in mind, individuals engage in other activities (named the ongoing task), distinguishing PM from simple planning.

Once the prospective component has been correctly detected, participants engage memory retrieval processes to recall what has to be done (i.e., retrospective component). These processes are influenced by the strength of the link between the prospective cue and the retrospective component. Indeed, according to reflexive associative theory [17,18,19], if the prospective cue and the action to perform are strongly associated, the mere perception of the cue reflexively triggers the retrieval of the intention [17,19,20]. By contrast, if there is only a weak link between the prospective cue and the content of the intention, a strategic memory retrieval process is engaged and may lead to poorer performance [20].

PM is generally thought to be impaired in ageing [16,21,22,23,24], although this effect has also been described as paradoxical. Older adults can indeed achieve performances equivalent to those of young individuals when the PM task relies on automatic processes, but when the PM task entails controlled attentional processes, performances are generally impaired. However, this effect can also be influenced by the conditions in which the task is administered (natural conditions vs. laboratory paradigms) as well as by the participant’s familiarity with the task and the environment [25,26]. Thus, an age-related decline in PM was observed using laboratory tasks, but older adults outperformed their young counterparts in natural conditions [27,28]. This age–PM paradox seems to be the consequence of a lack of experimental control in naturalistic settings and a lack of ecological validity for laboratory tasks [29]. The emergence of virtual reality in the field of neuropsychology may help to circumvent the biases of classical assessments. The use of virtual environments may be a good compromise, as it allows complex naturalistic situations to be reproduced, all the while maintaining a high level of experimental control [30]. In a recent study by our group, we exposed young and older individuals to a virtual town and asked them to recall EB and TB intentions. For EB intentions, the semantic link between the prospective and retrospective components was either weak or strong. While age-related PM decline affected the recall of both prospective and retrospective components, the recall of the latter seemed more challenging for older individuals when the link was weak. Thus, PM appears to be sensitive to ageing, even when the task is thought to be ecological [23].

Studies of sleep-dependent consolidation of retrospective episodic memories in older participants have yielded discrepant results. Some studies have shown that this process is impaired [31,32,33,34,35] and may be explained by changes in sleep architecture, especially the well-documented age-related decrease in SWS or changes in spindle density [36]. By contrast, other studies reported that sleep-dependent memory consolidation is preserved [37], especially when the material to learn is personally relevant [38]. As for PM, in a sample of older adults, Cavuoto et al. [39] assessed whether actigraphy-derived measures of sleep quality predicted performance on various memory tasks, including a PM task consisting in pressing the actimeter button twice a day. The authors showed that longer wake after sleep onset (WASO), longer sleep onset latency (SOL), and longer total sleep time (TST) predicted poorer performance on retrospective and working memory tasks. Surprisingly, PM performance was not related to sleep. The fact that part of the PM task was performed under sleep inertia, a transitory state of lowered arousal occurring immediately after awakening and resulting in a temporary decrement in subsequent performance [40], may explain, at least to some extent, the lack of a relationship between sleep and PM. More recently, Fine et al. [41] reported that sleep disruption (in the form of longer nocturnal awakenings) was associated with poorer PM performance on a laboratory task and mediated the association between age and PM performance. Finally, using polysomnography recordings, Scullin et al. [42] showed that PM consolidation is impaired in older adults, and this effect is mediated by a decrease in rapid eye movement (REM) sleep duration, contrasting with some results reported in young adults and highlighting the role of SWS [9]. The role of sleep in PM consolidation is therefore not yet fully understood.

In this context, our study was designed to investigate the effect of retention intervals filled with either sleep or daytime wakefulness on the recall of EB and TB intentions in young and older participants. We designed a PM task implemented in a complex virtual environment intended to optimize the ecological validity of the PM assessment. We also manipulated the strength of the semantic link between the prospective component and corresponding retrospective component to determine whether sleep preferentially strengthens congruent intentions rather than incongruent ones.

We expected to observe an effect of age on PM, especially the prospective component that is subserved by executive processes and frontal areas [43,44]. We further expected this effect to be more pronounced for TB intentions than EB ones [45,46,47]. We also hypothesized that sleep would preferentially reinforce the retrospective component of intentions, which is mainly hippocampus-dependent [48]. Based on memory schema theory according to which encoding and recalling information can be facilitated by prior related knowledge [49], we postulated that sleep would benefit the consolidation of intentions, especially when there was a strong semantic link between the prospective and retrospective components (i.e., congruent intentions). Finally, as some studies have reported impaired sleep-dependent memory consolidation in ageing [31,33,34], we expected sleep to be less beneficial for PM in older participants.

## 2. Materials and Methods

### 2.1. Participants

Participants were 28 young university students (13 men, mean age = 21.8 years, standard deviation (*SD*) = 2.8, range = 18–34) and 27 older adults (14 men, mean age = 62.7 years, *SD* = 4.7, range = 55–72). This study was approved by the regional ethics committee (Comité de Protection des Personnes Nord Ouest III) and written informed consent was obtained from each participant after a detailed presentation of the study. Participants’ characteristics are set out in Table 1.

All participants were native French speakers, right-handed, with normal or corrected-to-normal vision, and in good health, as attested by a medical examination. None of the participants were on medication during the study, except for oral contraceptives. Of the 55 participants, 53 had a Pittsburgh Sleep Quality Index (PSQI; [50]) below or equal to 5, indicating no sleep disturbances. Two older participants had PSQI scores of 6 or 7, but an interview with a sleep expert neurologist confirmed the absence of serious sleep difficulties. Scores on Parts A and B of the State-Trait Anxiety Inventory (STAI; [51]), reflecting state and trait anxiety, indicated that participants were not or mildly anxious. None of the participants had worked night shifts during the preceding year. All participants had normal scores on the 21-item Beck Depression Inventory (BDI; [52]; score ≤ 8). Extreme chronotypes (scores < 32 or > 70 on the morningness–eveningness questionnaire [53]) were excluded. The absence of signs of cognitive decline in the group of older adults was confirmed by the Mini Mental State Examination (MMSE; [54]; scores > 27). All volunteers followed a constant sleep schedule (according to their own sleep-wake schedule ± 1 hour) throughout the course of the study (4 weeks from the medical examination to the final testing session). Participants received financial compensation for their participation.

### 2.2. General Procedure

**Figure 1:**
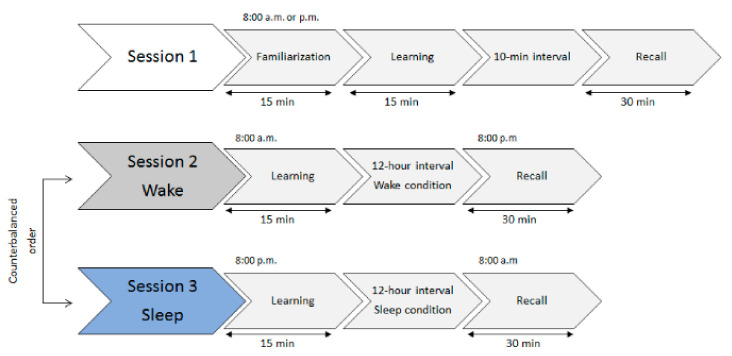
Study protocol. All participants came to the laboratory for three sessions, separated by at least 7 days. The first session included a familiarization phase with the virtual environment, followed by a learning phase and short-term (10 min) recall of intentions. This session was performed either in the morning or in the evening to control for a potential circadian confound. Participants then performed two further sessions in the wake and sleep conditions (counterbalanced order). In the sleep session, participants had a regular night of sleep at home. Wrist actigraphy was used to control for the absence of naps in the wake session and compliance with the sleep-wake schedule for the sleep condition.

The study comprised three sessions. On their arrival in the laboratory, participants first completed the Karolinska Sleepiness Scale (KSS; [55]) to measure their level of alertness before each experimental session. Participants were first immersed in the virtual environment to practice using the controller. The first session started with a familiarization phase during which participants visited the entire virtual museum. They were then asked to learn a series of 12 intentions (see Section 2.3.b for more details). Retrieval was assessed after a 10-min delay (10-min condition). One group of subjects (14 young and 14 older adults) performed this session at 8 a.m. (morning condition) and the other (14 young and 13 older adults) at 8 p.m. (evening condition). This first session was used to control for a potential circadian confound, as the learning and recall of intentions would take place at different times of the day in the sleep and wake sessions (see Figure 1).

In the second and third sessions, recall of intentions was probed after a 12-hour interval filled with either daytime wakefulness (wake condition) or nighttime sleep (sleep condition). In the wake condition, participants encoded the 12 intentions at 8 a.m. and recalled them at 8 p.m. (see Figure 1). In the sleep condition, intentions were encoded at 8 p.m. and recalled the following morning at 8 a.m. Volunteers took part in all three sessions in a within-subject design, where the order of the sleep and wake sessions was counterbalanced across participants. All the sessions were performed at Caen University Hospital, with at least one week between sessions to minimize the risk of interference.

### 2.3. Prospective Memory Task

#### 2.3.1. Virtual Environment

**Figure 2:**
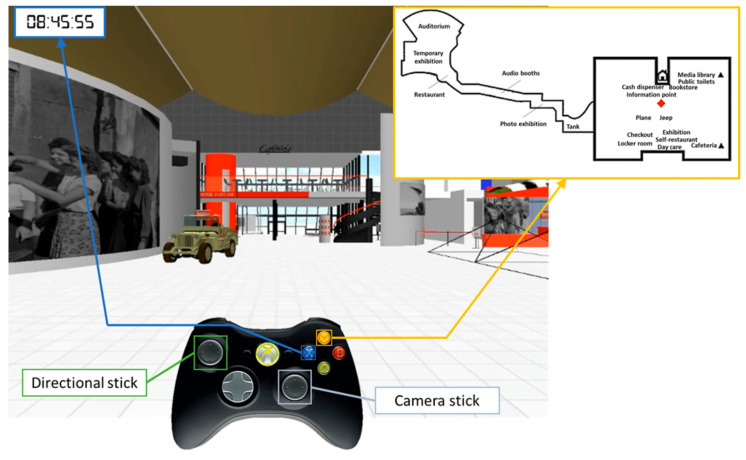
Representation of the virtual environment. This environment recreated the ground floor of the Memorial peace museum located in Caen, which is dedicated to World War II. Participants interacted with the virtual environment from a first-person perspective using a USB-wired controller with two joysticks and two buttons. The left joystick controlled the movements and the right joystick-controlled camera. The X and Y buttons enabled participants to view a clock and a map of the environment. These remained accessible throughout the exploration. The clock displayed a virtual time that served as the prospective cue for TB intentions. The map represented the virtual environment, showing with the names of relevant locations and the participant’s position.

PM was assessed using a complex virtual environment designed by the Interdisciplinary Center for Virtual Reality (CIREVE; Caen University, France) and developed with Unity 4.3 software (www.unity3d.com; Unity Technology, San Francisco, California, U.S.). This environment recreated the ground floor of the Memorial peace museum in Caen, which is dedicated to World War II (Figure 2). The virtual museum was displayed using a beamer on a 244-cm widescreen. Each participant was tested individually, after being comfortably seated approximately 400 cm from the widescreen. Participants were asked to report any feeling of faintness induced by the virtual reality or by the stress of performing the experiment. Participants interacted with the virtual environment from a first-person perspective using a USB-wired controller with two joysticks and two buttons. The left joystick allowed them to move around in the environment, and the right joystick controlled head movements and visual field orientation. The two buttons (X and Y) enabled participants to view a clock and a map of the environment, which remained accessible throughout the exploration. The clock displayed a virtual time that served as the prospective cue for TB intentions. The map represented the virtual environment, showing the names of relevant locations and the participant’s position. Finally, in PM studies, ongoing tasks are classically categorization or mental arithmetic calculation tasks, which are not close to everyday life situations. Accordingly, to increase the ecological validity of our paradigm, the ongoing task consisted here in viewing a series of 15 black and white pictures representing several striking events of World War II and displayed on the walls of the virtual environment. Participants were instructed to look at the pictures while verbally recalling the different intentions at the appropriate moment. There was no explicit instruction to memorize the pictures as when one is visiting a museum. When all the intentions had been recalled, we administered a recognition test featuring the 15 previously seen pictures, intermixed with 15 new ones. A different set of 15 pictures was used for each session (10 minutes, wake and sleep conditions).

#### 2.3.2. Encoding, Storage, and Recall of Intentions

• Encoding

In each session, the PM task consisted of the encoding and recall of 12 intentions presented on the screen of a laptop. Each intention was described through a unique sentence. The first part of the sentence contained the prospective cue (e.g., “At the cafeteria …”) and the last part contained the retrospective component, namely the action that participants had to execute (e.g., “… order a black coffee”). To ensure that all intentions were correctly encoded, a cued recall test was administered after learning, presenting the prospective component of each intention and asking participants to recall the retrospective one. Feedback was given after each response, and the procedure was repeated until subjects correctly recalled each intention twice in a row.

Among the 12 intentions to learn, four were TB intentions (e.g., “At 8:52 a.m., drop your bag off in the cloakroom”). For the eight EB intentions, half were semantically congruent (EB-link), featuring an obvious link between the prospective cue and the retrospective component (e.g., “At the cafeteria, order a black coffee”), whereas the others were incongruent (EB-nolink; e.g., “At the restaurant, buy the film screening program”).

• Storage

In the first session, the 10-min interval was filled by the completion of the STAI Parts A and B [51] measuring state and trait anxiety respectively. In the second and third sessions, participants left the laboratory after learning the 12 intentions and came back for the retrieval phase 12 hours later. This period was filled either by regular sleep at home (sleep condition) or by an equivalent period of daytime wakefulness during which participants were engaged in their usual activities and were instructed not to take naps (wake condition). Compliance with the sleep–wake schedule and the absence of naps were assessed using wrist actigraphy (Actiwatch; Cambridge Neurotechnology Ltd). Participants wore the actigraph solely during the retention intervals (one night for sleep condition and one day for wake condition). Furthermore, upon their return to the laboratory for the retrieval phase, participants completed the KSS and the St Mary’s Hospital Sleep Questionnaire [56] to assess the quality of their sleep during the previous night.

• Recall

Recall of intentions was probed after a 10-min or 12-hour interval. Before each session, participants were informed that they would be immersed again in the virtual environment and that they would have to look at a series of pictures, while executing several actions at the appropriate moment. In the sleep condition, retrieval was tested at least 45 min after awakening in order to minimize any effect of sleep inertia on memory retrieval. For each session, participants were again immersed in the virtual museum where they had to recall the sentences describing the intentions as accurately as possible and at the appropriate moment. Each session lasted at least 15 min and participants were allowed to stay for no more than 20 min.

A maximum of 6 points could be awarded for each intention depending on the quality of recall. The scoring method is detailed in Table 2. Thus, for the prospective component of EB intentions, 2 points were awarded if recall occurred when the participant entered the place cueing retrieval for the first time, 1 point if it occurred after the second time, and 0.5 point if it occurred when the participant entered the place for at least the third time. A maximum of 2 points were also awarded for correct recall of the retrospective component: 2 points for the exact action, 1 point for a closely related action, and 0.5 point for an incomplete action. For the prospective component of TB intentions, 2 points were awarded if the intention was recalled at the exact time, 1 point if it was recalled within 1 minute of the target time, and 0.5 point if it was recalled within 2 minutes of the target time. As for the retrospective component, 2 points were awarded for the exact action, 1 point for a closely related action, and 0.5 point for an incomplete action. Finally, for both EB and TB intentions, 2 points were awarded for a correct association between the prospective and retrospective components (*associative component*), and 0 for an incorrect association (see Table 2 for examples). We calculated an overall PM score, and defined as the sum of scores for the retrieval of prospective, retrospective, and associative components.

### 2.4. Actigraphy Data Analysis

Actigraphy data recorded during the post-learning night were analyzed using MotionWare software (version 1.1.25; CamNTech Ltd., Cambridge, UK) and sampled using a 5-second epoch. A sensitivity threshold of 20 counts was applied to distinguish activity from rest. “Lights off” and “Got up” markers were placed in the appropriate position according to the event markers placed by the participant and cross-validated with the light sensor data and information from a sleep diary. “Fell asleep” and “Woke up” markers were automatically adjusted according to a combination of the activity data and the “Lights off” and “Got up” markers, allowing us to measure assumed TST in minutes, SOL (min), WASO (min), and sleep efficiency (SE %). Finally, a fragmentation index was calculated as the sum of the percentages of mobile time and immobile bouts lasting no more than 1 minute during the assumed sleep period.

### 2.5. Statistical Analyses

Demographical, behavioral and actigraphy data were compared between young and older participants using one-way analyses of variance (ANOVA), except sex ratio, for which we used a Chi² test. As for PM scores, statistical analyses consisted of mixed analyses of variance (ANOVA) followed by post hoc Tukey honestly significant difference tests where applicable. Pearson’s correlation analyses were also performed to search for associations between actigraphy-derived sleep parameters and PM performance. All analyses were performed using Statistica software (version 13.4.0.14; Statsoft, Tulsa, OK).

## 3. Results

Participants’ characteristics are reported in Table 1. No differences were found between the two groups in terms of sex ratio (*p* = 0.69), education level (*p* = 0.92), anxiety (STAI-B; *p* = 0.75), depression (BDI, *p* = 0.19), or PSQI score (*p* = 0.7). The MMSE scores of older adults were within the normal range.

A visual inspection of actigraphy recordings confirmed compliance to the sleep–wake schedule and the absence of naps, for each subject, in the sleep and wake sessions, respectively. Comparisons of sleep parameters obtained during the post-learning night showed that there were no group differences in terms of TST, SOL, WASO, SE, or sleep fragmentation (all *p* values > 0.25; Table 1).

ANOVAs with group (young vs. older) as between-subject factor were performed on the number of trials needed to correctly recall the intentions twice, for each separate condition (EB-link, EB-nolink, and TB). For EB-link intentions, the ANOVA failed to reveal a significant effect of Group (*F*(1,53) = 1.79, *p* > 0.18). By contrast, for EB-nolink and TB intentions, a significant effect of Group was observed (EB-nolink: *F*(1,53) = 13.1, *p* < 0.001; TB: *F*(1,53) = 8.69, *p* < 0.01), indicating that for these two types of intentions, older adults needed more trials than young ones to correctly encode the intentions (Table 3).

Table 4 provides detailed information about participant’s behavioral performance in the 10-min condition, distinguishing participants who performed this session in the morning from those who were tested in the evening. Table 5 shows behavioral performance of each group in the wake and sleep conditions according to type of intention and PM component.

### 3.1. Effect of Time of Testing on Subjective Sleepiness and PM Performance

In order to ensure that the potential difference in PM performance between the sleep and wake sessions could not be attributed to the time of day at which participants learned the intentions and were tested (8 a.m. or 8 p.m.), we first ran a mixed ANOVA with both group (young vs. older) and testing time (8 a.m. vs 8 p.m.) as between-subject factors on KSS scores obtained in the 10-min condition. This analysis did not reveal a significant main effect of either group (*F*(1, 51) = 0.07, *p* > 0.79) or testing time (*F*(1, 51) = 0.66, *p* > 0.41), and there was no interaction between these factors (*F*(1, 51) = 3, *p* > 0.08), indicating that the level of alertness did not differ between groups or according to time of testing.

We then conducted a similar analysis on overall PM performances (corresponding to the sum of scores for the prospective, retrospective, and associative components) in the 10-min condition. This analysis revealed no significant effect of either group (*F*(1, 51) = 0.09, *p* > 0.76), or testing time (*F*(1, 51) = 0.10, *p* > 0.74), and there was no interaction between these two factors (*F*(1, 51) = 0.29, *p* > 0.59), indicating that the recall of intentions after a 10-min interval did not differ between young and older participants, or according to the time of testing (8 a.m. vs. 8 p.m.). Analyses considering the three types of intentions and components separately revealed the same pattern of results (data not shown).

Thus, PM performance did not differ either between young and older participants, or according to time of testing.

### 3.2. Effects of Sleep and Wakefulness on the Consolidation and Recall of EB and TB Intentions

To investigate the potential benefit of sleep on PM, we then performed mixed ANOVAs on each type of intention (EB-link, EB-nolink, and TB) with condition (wake vs. sleep) as within-subject factor and group (young vs. older) as between-subject factor.

**Figure 3:**
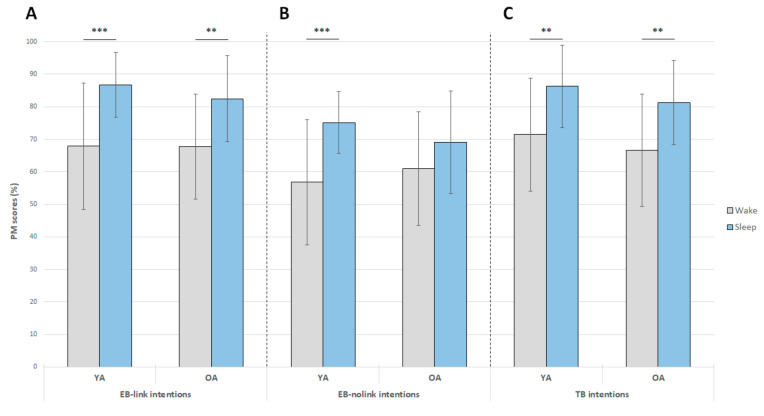
Effect of 12-h retention intervals filled with either daytime wakefulness or sleep on the recall of EB-link, EB-nolink and TB intentions (expressed as mean performance ± standard error of the mean (*SEM*)) in young and older adults (YA and OA, respectively). **: *p* < 0.01; ***: *p* < 0.001.

For EB-link intentions, this analysis revealed no significant effect of group (*F*(1, 53) = 0.27, *p* > 0.60), but there was a significant effect of condition (*F*(1, 53) = 30.6, *p* < 0.001) in favor of better performance after sleep than after wakefulness (Figure 3A). The group x condition interaction was not significant (*F*(1, 53) = 0.5, *p* > 0.47). 

For EB-nolink intentions, a similar analysis revealed no significant effect of group (*F*(1, 53) = 0.04, *p* > 0.84), but there was a significant effect of condition as described above (*F*(1, 53) = 20.1, *p* < 0.001; Fig. 3B). A trend was observed for the group x condition interaction (*F*(1, 53) = 3.1, *p* = 0.084). Post hoc comparisons indicated that in young participants, recall of EB-nolink intentions was better after sleep than after an equivalent period of wakefulness (*p* < 0.001). This benefit of sleep was not observed in older adults (*p* > 0.23; Figure 3B). In addition, in each condition (wake, sleep), young and older participants had equivalent performance (wake: *p* > 0.85; sleep: *p* > 0.67).

For TB intentions, the ANOVA failed to reveal a significant effect of group (*F*(1, 53) = 1.29, *p* > 0.26) but there was a significant effect of condition (*F*(1, 53) = 27.3, *p* < 0.001). Recall of TB intentions was better after sleep than after wakefulness (Figure 3C). The group x condition interaction was not significant (*F*(1, 53) = 0.001, *p* > 0.97).

To sum up, there was no effect of age on the retrieval of the different types of PM intentions. On the whole, recall was better after sleep than after an equivalent period of wakefulness across both groups and all types of intentions, except for EB-nolink intentions (no benefit of sleep on this type of intentions in older participants).

### 3.3. Effects of Sleep and Wakefulness on the Consolidation and Recall of Prospective, Retrospective, and Associative Components of PM

We also investigated the effect of sleep on the consolidation and recall of the three components of PM (i.e., prospective, retrospective, and associative components). To this end, we conducted mixed ANOVAs on each PM component with condition (wake vs. sleep) as within-subject factor and group (young vs. older) as between-subject factor.

**Figure 4:**
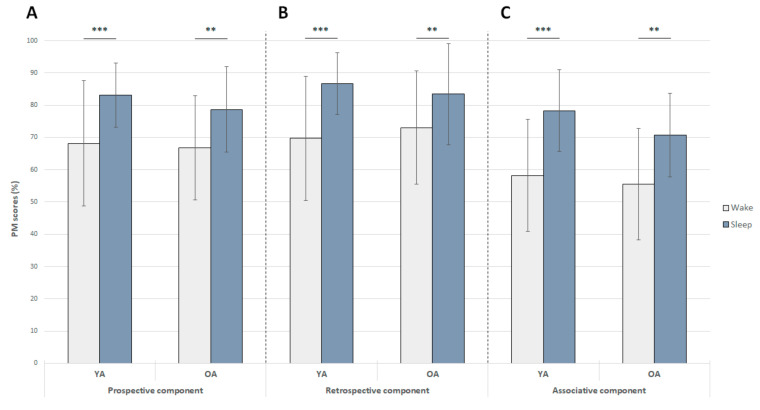
Effect of 12-h retention intervals filled with either daytime wakefulness or sleep on the recall of the prospective, retrospective, and associative components of intentions (expressed as mean performance ± standard error of the mean (SEM)) in young and older adults (YA and OA respectively). **: *p* < 0.01; ***: *p* < 0.001.

For the prospective component, this analysis failed to reveal a significant effect of group (F(1, 53) = 0.78, *p* > 0.38), but there was a significant effect of condition (F(1, 53) = 34.8, *p* < 0.001). Recall of the prospective component of intentions was better after sleep than after wakefulness (Figure 4A). The group x condition interaction was not significant (F(1, 53) = 0.5, *p* > 0.48).

For the retrospective component, the ANOVA failed to reveal a significant effect of group (F(1, 53) = 0, *p* > 0.99), but there was a significant effect of condition as described above (F(1, 53) = 34.8, *p* < 0.001; Figure 4B). A trend was observed for the group x condition interaction (F(1, 53) = 2.98, *p* = 0.09). Post hoc comparisons revealed that the recall of the retrospective component by both young and older adults was better after sleep than after wakefulness (young participants: *p* < 0.001; older participants: *p* < 0.01; Figure 4B). Recall performance did not differ between young and older participants in the wake (*p* > 0.83) or sleep (*p* > 0.84) conditions.

For the associative component, the analysis failed to reveal a significant effect of group (F(1, 53) = 1.30, *p* > 0.26), but there was an effect of condition as previously mentioned (F(1, 53) = 45.7, *p* < 0.001; Figure 4C). The group x condition interaction was not significant (F(1, 53) = 0.9, *p* > 0.34).

To sum up, there was no effect of age on the recall of the three PM components, but a significant effect of sleep as performance was better after sleep than after wakefulness in both groups.

### 3.4. Performance on the Ongoing Task

We also analyzed performances obtained on the ongoing task by running a mixed ANOVA on recognition accuracy scores (d’), reflecting the ability to correctly recognize old items and reject new ones, with group (young vs older) as between-subject factor and condition (wake vs. sleep) as within-subject factor. This analysis revealed that the effect of group tended toward significance (F(1, 53) = 3.82, *p* = 0.06), but there was no effect of condition (F(1, 53) = 1.96, *p* > 0.75), and no interaction effect (F(1, 53) = 1.96, *p* > 0.16), indicating that recognition accuracy was lower for older participants than for young ones, in both sleep and wake conditions (Table 5).

### 3.5. Associations between Objective Measures of Sleep and PM

Finally, we investigated the link between PM performance and actigraphy-derived sleep parameters for the sleep condition. To do so, we computed correlation analyses between PM performance and each sleep parameter (TST, SOL, WASO, SE, and sleep fragmentation), for the whole sample as well as for each of the two groups. None of these parameters were correlated with PM performance (data not shown).

## 4. Discussion

The aim of the present study was to investigate the effect of retention intervals filled with sleep or daytime wakefulness on the recall of intentions in young and older participants. A short-term condition (recall after 10 min) was added to control for potential circadian effects, as participants did not learn and were not tested at the same time of day in the sleep and wake conditions. Analysis of performances in this 10-min condition revealed that overall PM performance was similar across young and older participants, whatever the time of testing. This result confirmed that baseline performance was not influenced by a possible difference in the level of alertness due to the time of day.

### 4.1. Effects of Sleep on PM

Young adults had better recall performances after sleep than after an equivalent period of wakefulness for all three types of intentions we tested. Interestingly, sleep reinforced not only intentions whose content was semantically related to the prospective cue (EB-link), but also intentions where there was only a weak link between the prospective cue and the action to perform (EB-nolink). These results are reminiscent of the debate about the impact of encoding depth—or, more broadly, task difficulty—on memory consolidation, but also about the extent to which the recall of semantically related and unrelated word pairs benefits from sleep. As for encoding depth or task difficulty, Schmidt et al. [57] assessed the consolidation of concrete versus abstracts nouns and concluded that changes in spindle activity were observed during napping after the difficult (abstract words) encoding condition but not after the easy one (more concrete nouns). Moreover, Drosopoulos et al. [58] varied the encoding strength by applying different learning criteria during the memorization of word-pair associates. They reported that the benefit of sleep was greater for weak than for strong associations. However, studies assessing the consolidation of semantically related and unrelated word pairs have yielded mixed findings. While some report sleep benefits on recall performance for word pairs that have a strong semantic association [59,60], others suggest that sleep instead benefits the processing of word pairs that are not semantically related [61,62,63]. To the best of our knowledge, the study by Payne et al. [64] is the only one that has directly compared the effects of sleep and wake retention intervals on the consolidation and recall of semantically related and unrelated word pairs. These authors reported that while 12 hours of wakefulness only had a detrimental effect on unrelated word pairs, sleep was beneficial for the consolidation of related and unrelated word pairs. Our results for congruent (EB-link) and incongruent (EB-nolink) intentions, which differed solely in the semantic strength of the association between the prospective cue and the action to perform, confirm, and extend these findings to PM.

The benefit of sleep on intentions presenting a strong link between the cue triggering retrieval and the content of the action can also be interpreted in the light of schema theory, whereby encoding and recalling information can be facilitated by prior related knowledge [65,66,67]. Memory schemas refer to cognitive structures that encode gist representations of repeatedly encountered events [66]. The repetition of similar episodes (e.g., repeatedly buying a coffee in a cafeteria) can contribute to the formation of an associative framework connecting core elements of these experiences. Schema formation and integration may benefit from sleep [68,69]. Previous studies have highlighted the role of both non-REM [70] and REM sleep [49] in these processes, depending on the type of memory schema targeted.

In the older adults, our data revealed a different effect of sleep on PM intentions, reinforcing congruent (EB-link) and TB intentions, but not incongruent (EB-nolink) ones. As discussed above, sleep has been shown to preferentially reinforce intentions that are coherent with prior knowledge. However, while this has been demonstrated in young adults [49,70], we do not know whether age-related changes in sleep, as well as in brain structure and function, still allow this preferential consolidation of congruent information to take place. Spindle activity has been associated with the integration of new and incongruent information with preexisting knowledge in young adults [71]. Sleep spindles are electrophysiological features of N2 sleep, and their density, amplitude, and duration are known to decrease in the course of ageing [72]. Given these elements, but despite the fact that we did not collect polysomnography data, we might expect an impairment of consolidation of incongruent intentions in older adults. This hypothesis would also imply that sleep benefits less TB intentions. This was not the case. We can surmise that the association between a schedule and an action was easier to memorize, and probably closer to everyday life situations, than the two components of EB-nolink intentions. We nevertheless acknowledge that this is purely speculative and requires further investigation.

We also examined the effect of sleep on the retrieval of the prospective and retrospective components of PM. Although sleep had a significant effect on each component in both groups, the statistical trend we observed for the interaction between group and the retrospective component led to foresee a differential effect of sleep in young versus older participants. Such an effect would have been explained by the fact that the retrospective component of PM engages episodic memory, which is one of the memory systems most vulnerable to the effects of age [73]. This component also relies on the hippocampus [74], which is also known to undergo structural and functional changes in ageing (for reviews, see [73,75,76]). Further investigations concerning this particular component of PM are needed in order to understand the absence of obvious differences between groups and conditions.

In the present study, we decided to add an associative component to check that participants executed the correct action in response to the corresponding cue and to avoid confusions between intentions. This latter component relies on binding processes that are known to be impaired in ageing [77,78,79] and involved in PM [21,23]. Despite that, recall of this associative component was equivalent between young and older participants.

### 4.2. Effects of Age on PM

One of the most robust results of our study was the absence of age effects on PM performance, whatever the nature of the retention interval and the type of intention. These findings raise the question of the potential impact of cognitive reserve on sleep and memory. The older participants included in our study were highly educated, education being a common proxy of cognitive reserve, and involved in many activities [80]. The impact of cognitive reserve on sleep quality has seldom been investigated. In a study among nondemented older adults, Zimmerman et al. [81] found that individuals with a lower education level tended to be more vulnerable to the negative effects of sleep onset/maintenance difficulties on some cognitive tasks such as verbal fluency. In addition, cognitive reserve may also have a protective effect against cognitive deficits associated with sleep apnea [82], a sleep disorder that is frequently observed in the ageing population. We can postulate that individuals with a high cognitive reserve are better able to compensate for neural dysfunction due to disturbed sleep and may maintain good memory performance.

Based on this idea, another explanation for the similar PM performances of the young and older adults concerns sleep quality. The latter, which was assessed using actigraphy, did not differ between the two groups in terms of either TST, SOL, WASO, or fragmentation. Actigraphy is not the best tool for assessing sleep, so we cannot definitively conclude that sleep quality was not reduced in older participants, especially since age-related changes in sleep architecture and microstructure are well documented (for a review, see [36]). Ageing brings a decrease in the amount of SWS, the deepest non-REM sleep stage, starting from the fourth decade. REM sleep is also affected by ageing, but at a later stage in the ageing process and to a lesser extent than SWS [36,83]. In young adults, the successful implementation of delayed intentions mainly benefits from SWS [9]. However, Scullin et al. [42] recently found that REM sleep duration predicted PM performance in older adults. By contrast, slow oscillations and sleep spindles were not related to PM consolidation. Further studies featuring polysomnography are warranted to pinpoint the respective contributions of SWS and REM sleep to PM consolidation in ageing.

The lack of an age-related decline in PM performance can also be explained, at least in part, by the nature of our experimental task which was closer to everyday life situations than laboratory paradigms (“age prospective memory paradox” [25,84,85]). We used a realistic virtual environment reproducing a well-known museum located in Caen and dedicated to World War II. This environment was therefore quite familiar to participants, particularly the older ones who had been more directly concerned by these historical events than the younger individuals. We can therefore surmise that this familiar environment increased the motivation to perform the PM task, especially in older adults. Indeed, previous studies have shown associations between motivation and PM performance [86,87].

Furthermore, we noticed that the older adults needed more trials to learn the EB-nolink and TB intentions than the young ones did. This higher number of repetitions may have reinforced encoding in older adults, resulting in levels of consolidation and recall performance similar to those observed in the young individuals. A recent study showed that repeated rehearsal of information initiates memory consolidation, and that sleep is required to stabilize these changes [88]. Thus, repeated rehearsal and sleep jointly contribute to long-term memory consolidation. Accordingly, in older adults, the repetition of intentions strengthened encoding and initiated consolidation, and may have compensated for potential changes in sleep quality, that have yet to be objectified, leading to performance comparable to that of the young participants.

We did, however, observe an effect of age on the recognition task administered at the end of the session to check that participants correctly performed the ongoing task. The latter consisted of looking at a series of pictures, as in a museum. Classically, ongoing tasks take the form of categorization or mental arithmetic tasks, or even working memory tasks. Our choice was guided by the fact that these latter do not reproduce what happens in everyday life. We therefore used a task that did not particularly elicit controlled processes, during either the encoding or retrieval. Although encoding was incidental in the 10-min condition, it may not have been in the sleep and wake conditions. However, in these two cases, participants performed the task in the same conditions. We expected this encoding task not to interfere with the recall of intentions and to be accurately executed by both young and older adults. We observed that older participants had poorer recognition performances than young participants in both the wake and sleep conditions. As PM performance was equivalent in the young and older participants, we can surmise that this ongoing task required fewer cognitive resources than the tasks that are classically used, which may have masked the effect of age reported in previous studies [21,22,24]. Another possible explanation is that older adults focused on the recall of intentions, and paid little attention to the pictures, leading to poorer recognition performances.

### 4.3. Limitations

Despite its strengths, this study has several limitations. First, the ongoing task we administered did not correspond to those classically used in PM assessments, but it was chosen to mimic real-life situations as closely as possible. The recognition task administered after intention recall was an imperfect reflection of what occurred during the exploration of the environment, precisely because it was administered afterwards. Future studies should use a realistic ongoing task that does not elicit memory processes in order to confirm (or not) the effect we observed. Finally, some of the results reported here were only statistical trends, precluding any firm conclusion about the differential effect of sleep on PM intentions and components in ageing. These results should be interpreted with caution, pending replication.

## 5. Conclusions

This study was an initial attempt to improve the ecological validity of PM assessment as part of an investigation of sleep-dependent memory consolidation. To this end, we developed a graphically realistic virtual environment reproducing a museum. Intentions were created to reproduce the actions we are liable to perform in everyday life as closely as possible. The content of the intentions was much more rich and varied than in most previous studies. Moreover, this environment enabled us to achieve a level of experimental control similar to that of laboratory tasks. However, experimental control could be enhanced still further by refining the recording of participant’s behavior while performing the task. Recording physiological data (e.g., eye movements, changes in heart rate) would doubtless shed light on age-related behavioral changes and cognitive strategies. Technical advances will also contribute to the design of virtual environments that are even more complex and realistic, and probably also more immersive, with virtual reality glasses or a headset thus providing better support for the evaluation of PM and other cognitive functions in young adults and older adults.

Further studies using polysomnography are also needed to decipher the exact electrophysiological contribution of sleep to PM enhancement in older adults. Finally, this work also highlighted the need to better understand the impact of cognitive reserve on sleep quality, as this variable may modulate the association between sleep and memory performance.

## Figures and Tables

**Table 1:** Demographic, behavioral, and actigraphy data for young and older participants.

	Young Adults	Older Adults	p Value
	M ± SD	M ± SD	
**Age** (years)	21.8 ± 2.8	62.7 ± 4.7	<0.001 ***
**Sex ratio** (M/F)	13 / 15	14 / 15	0.69
**Education** (years)	13.6 ± 1.7	14.2 ± 2.7	0.35
**STAI-A**	28.9 ± 5.0	31.9 ± 9.0	0.13
**STAI-B**	34.5 ± 5.2	34.9 ± 8.1	0.86
**BDI**	1.7 ± 1.4	2.0 ± 2.0	0.51
****MMSE****	-	29.5 ± 0.6	-
**PSQI**	2.8 ± 1.4	2.7 ± 1.8	0.78
**Sleep parameters**			
TST (min)	377.6 ± 92.9	370.3 ± 63.1	0.74
SOL (min)	2.3 ± 6.7	3.3 ± 5.5	0.57
WASO (min)	66.4 ± 36.8	69.2 ± 26.2	0.75
SE (%)	84.3 ± 7.9	82.7 ± 9.5	0.53
Sleep Fragmentation	27.6 ± 10.8	32.8 ± 19.3	0.25

*Note*: M = mean; *SD* = standard deviation; STAI: State-Trait Anxiety Inventory (Part A: state anxiety; Part B: trait anxiety); BDI: Beck Depression Inventory; MMSE: Mini-Mental State Examination; PSQI: Pittsburgh Sleep Quality Index; TST: total sleep time; SOL: sleep onset latency; WASO: wake after sleep onset; SE: sleep efficiency.

**Table 2:** Scoring method for prospective memory intentions.

		POINTS	PM INTENTIONS		
			**EB-link intentions** *“At the media library, rent a DVD on the Normandy landings.”*	**EB-nolink intentions** *“At the restaurant, buy the movie program.”*	**TB intentions** *“At 05:58 p.m., take a picture of the tank.”*
**COMPONENTS**	Prospective	2	Recall occured when entering the media library for the first time	Recall occured when entering the restaurant for the first time	Recall occured at the exact time
		1	Recall occured when entering the media library for the second time	Recall occured when entering the restaurant for the second time	Recall occured within 1 minute of the target time
		0.5	Recall occured when entering the media library for the third or more time	Recall occured when entering the restaurant for the third or more time	Recall occured within 2 minutes of the target time
		0	The participant did not remember that something had to be done at the media library	The participant did not remember that something had to be done at the restaurant	Recall occured more than 3 minutes before or after the target time/The participant did not remember that something had to be done at 05:58 p.m.
	Retrospective	2	Action perfectly recalled	Action perfectly recalled	Action perfectly recalled
		1	“I have to rent a DVD on **World War II**”	“I have to buy the program **of the exhibition**”	“I have to take a picture of **the plane**”
		0.5	“I have to rent a DVD…”	“I have to buy a program…”	“I have to take a picture…”
		0	The action was not recalled	The action was not recalled	The action was not recalled
	Associative	2	Prospective and retrospective components recalled together	Prospective and retrospective components recalled together	Prospective and retrospective components recalled together
		0	Prospective and retrospective components recalled separately	Prospective and retrospective components recalled separately	Prospective and retrospective components recalled separately

*Note*: This table illustrates the scoring method for the retrieval of EB and TB intentions. Examples of incorrect elements during recall are shown in red.

**Table 3:** Number of trials required by young and older participants.

	Young Adults	Older Adults
EB-link intentions	7.7 ± 2.5	9.0 ± 2.7
EB-nolink intentions	9.8 ± 2.7	13.7 ± 2.8 ***
TB intentions	11.8 ± 2.6	14.6 ± 2.5 **

*Note:* This table shows the number of trials that participants needed to correctly recall each intention twice in a row, for each type of intention. It should be noted that for this analysis, data for the 10 min, sleep, and wake conditions were pooled. Data are presented as mean ± standard error of the mean (*SEM*). EB-link: intention with a strong link between the cue triggering retrieval and the action to perform; EB-nolink: intention with no link; TB: intention associated with a specific schedule. **: *p* < 0.01, ***: *p* < 0.001.

**Table 4:** Behavioral performance in the 10-min condition.

			Morning	Evening
**Young adults**		Ongoing task (d’)	2.4 ± 0.5	2.3 ± 0.9
	PM intentions	Global PM scores (%)	79.4 ± 9.2	78.7 ± 8.5
		EB-link (%)	84.5 ± 10.7	84.7 ± 11.3
		EB-nolink (%)	69.5 ± 13.6	73.8 ± 12.1
		TB (%)	84.1 ± 13.4	77.7 ± 14.7
	PM components	Prospective (%)	73.4 ± 11.7	72.9 ± 7.4
		Retrospective (%)	89.1 ± 6.0	93.6 ± 6.4
		Associative (%)	75.6 ± 12.5	69.6 ± 14.5
**Older adults**		Ongoing task (d’)	2.0 ± 0.7	2.3 ± 0.5
	PM intentions	Global PM scores (%)	76.8 ± 11.4	79.4 ± 8.6
		EB-link (%)	86.2 ± 9.6	84.1 ± 9.1
		EB-nolink (%)	71.6 ± 17.7	73.4 ± 12.5
		TB (%)	72.6 ± 14.4	80.8 ± 17.6
	PM components	Prospective (%)	67.3 ± 13.8	73.6 ± 10.8
		Retrospective (%)	91.1 ± 7.2	93.6 ± 6.9
		Associative (%)	72.0 ± 15.6	71.2 ± 11.7

*Note:* This table distinguishes between participants who were tested in the morning and those who were tested in the evening. Data are presented as mean ± standard error of the mean (*SEM*). EB-link: intention with a strong link between the cue triggering retrieval and the action to perform; EB-nolink: intention with no link; TB: intention associated with a specific schedule.

**Table 5:** Behavioral performance in the wake and sleep conditions.

			Wake	Sleep
**Young adults**		Ongoing task (d’)	2.7 ± 0.7 ^#^	2.5 ± 0.8
	PM intentions	Global PM scores (%)	65.4 ± 15.9	82.7 ± 8.4 ***
		EB-link (%)	67.9 ± 19.5	86.8 ± 9.9 ***
		EB-nolink (%)	56.8 ± 19.2	75.1 ± 9.5 ***
		TB (%)	71.4 ± 17.4	86.2 ± 12.7 **
	PM components	Prospective (%)	68.2 ± 15.7	83.2 ± 8.5 ***
		Retrospective (%)	69.7 ± 15.0	86.7 ± 6.8 ***
		Associative (%)	58.2 ± 18.6	78.3 ± 12.8 ***
**Older adults**		Ongoing task (d’)	2.1 ± 0.8	2.2 ± 0.5
	PM intentions	Global PM scores (%)	65.1 ± 10.6	77.6 ± 10.6 ***
		EB-link (%)	67.8 ± 16.2	82.4 ± 13.2 **
		EB-nolink (%)	61.0 ± 17.5	69.1 ± 15.7
		TB (%)	66.6 ± 17.2	81.3 ± 13.0 **
	PM components	Prospective (%)	66.8 ± 8.6	78.6 ± 11.4 **
		Retrospective (%)	73.1 ± 13.2	83.4 ± 10.9 **
		Associative (%)	55.6 ± 14.2	70.7 ± 15.3 **

*Note:* Data are presented as mean ± standard error of the mean (*SEM*). EB-link: intention with a strong link between the cue triggering retrieval and the action to perform; EB-nolink: intention with no link; TB: intention associated with a specific schedule. #: *p* = 0.097 for the group (young vs. older) comparison; **: *p* < 0.01 and ***: *p* < 0.001 for comparisons between the wake and sleep conditions.

## Abbreviations

ANOVA	Analysis of variance
BDI	Beck Depression Inventory
EB	Event-based
KSS	Karolinska Sleepiness Scale
MMSE	Mini-Mental State Examination
PM	Prospective memory
PSQI	Pittsburgh Sleep Quality Index
REM	Rapid Eye Movement
SD SE	Standard deviation Sleep Efficiency
SEM SOL	Standard error of the mean Sleep Onset Latency
STAI	State-Trait Anxiety Inventory
SWS	Slow-wave sleep
TB TST WASO	Time-based Total Sleep Time Wake After Sleep Onset
